# Effect of Exercise on Chronic Tension-Type Headache and Chronic Migraine: A Systematic Review

**DOI:** 10.3390/healthcare13131612

**Published:** 2025-07-04

**Authors:** Cindy Johana Palacio-Del Río, Sofía Monti-Ballano, María Orosia Lucha-López, César Hidalgo-García, José Miguel Tricás-Moreno

**Affiliations:** Unidad de Investigación en Fisioterapia, Spin off Centro Clínico OMT-E Fisioterapia SLP, Universidad de Zaragoza, Domingo Miral s/n, 50009 Zaragoza, Spain; 782514@unizar.es (C.J.P.-D.R.); smonti@unizar.es (S.M.-B.); jmtricas@unizar.es (J.M.T.-M.)

**Keywords:** chronic pain, central sensitization, tension-type headache, migraine, exercise

## Abstract

**Objectives:** This study aims to identify the effectiveness of exercise in chronic tension-type headache and chronic migraine. **Methods:** The PICOS (Population, Intervention, Comparator, Outcomes, Study design) strategy was followed, where P—patients with chronic tension-type headache or chronic migraine; I—exercise; C—conventional treatment; O—pain reduction; and S—RCTs (randomized controlled trials) and quasi-experimental trials. Studies with a high risk of bias according to the RoB 2 (Risk of Bias) scale and with a score < 6 according to the PEDro (Physiotherapy Evidence Database) scale were excluded. The PRISMA (Preferred Reporting Items for Systematic Reviews and Meta-Analyses) statement was followed. The databases Pubmed, Web of Science, and Scopus were searched in November 2024. The PEDro and RoB2 scales were used to assess the risk of bias and internal validity of the studies. The results were synthesized qualitatively. **Results:** Ten studies with a total sample of 848 subjects were analyzed, comparing therapeutic interventions with an exercise versus conventional treatment. In most of the studies, the exercise group significantly outperformed the control group in pain reduction. **Discussion:** The findings suggest that exercise improves central pain modulation and reinforces the potential of body strategies such as postural integration. The main limitations found were the limited evidence on exercise’s role in chronic tension-type headache or migraine and the risk of bias due to the difficulty of blinding patients, therapists, and evaluators. **Conclusions:** The studies analyzed have shown that exercise could be an effective strategy to support the management of chronic tension-type headache and migraine.

## 1. Introduction

Headaches are a leading cause of disability worldwide, with migraine and tension-type headache (TTH) being the most prevalent primary forms. These conditions impose significant costs on healthcare systems and diminish the quality of life for millions. In Europe, the prevalence of TTH is a staggering 62.6% [1]. Recent studies suggest that the prevalence of migraine in the global population is around 14–15% [2]. The Global Burden of Disease study ranks migraine and TTH as the second leading cause of years lived with disability globally [1,3,4].

The International Headache Society defines chronic tension-type headache (CTTH) as a disorder characterized by frequent episodes of pain, lasting from minutes to days. Symptoms typically include a mild to moderate, oppressive, or tightening pain that is usually bilateral and may be accompanied by mild nausea, photophobia, or phonophobia, without worsening with routine physical activity [5,6]. CTTH occurs 15 or more days per month for over three months, or at least 180 days per year. CTTH significantly impacts an individual’s quality of life and leads to considerable disability [4,5]. A common diagnostic challenge arises in differentiating TTH from migraine due to their frequent similar phenotypic presentations, which stem from overlapping pathophysiological mechanisms [1,5]. For migraine to be classified as chronic, there must be at least 15 days per month with painful episodes [5,7].

Migraine can be unpredictable, and in 2.5% of undiagnosed cases, it can progress to a chronic disease, leading to serious long-term effects. This underscores the need for early and appropriate interventions to prevent symptom worsening. Consequently, there is a growing emphasis on identifying evidence-based non-pharmacological approaches that can complement pharmacotherapy in migraine prevention [8,9,10,11,12].

Central pain or central sensitization syndrome (CSS) originates from prolonged peripheral noxious stimuli that, over time, become established in the central nervous system (CNS). This leads to a state where even minimal nociceptive input can trigger widespread sensitization. Key indicators of central sensitization (CS) include generalized hyperalgesia (increased pain sensitivity), allodynia (pain from normally non-painful stimuli), absence of conditioned pain modulation (a deficit in the body’s natural pain-inhibitory mechanisms), and sleep disturbances. All these characteristics are observed in patients with migraine or TTH, particularly in their chronic forms [3,13,14,15,16,17]. CS helps explain “non-organic” symptoms, which manifest as an abnormal increase in pain due to neuronal hyperexcitability and dysfunction in the CNS’s descending and ascending pathways. These physical symptoms often lack a specific organic cause and are frequently associated with high rates of psychopathology, predicting poorer treatment outcomes [13,18].

Therefore, for patients whose conditions are influenced by central pain mechanisms—such as those with fibromyalgia, chronic fatigue syndrome, irritable bowel syndrome, temporomandibular joint disorder, migraine, and TTH (all categorized as disorders linked to the CSS)—treatment strategies should aim to normalize CNS sensitization. This includes selecting treatments that integrate physical, psychological, or cognitive and educational approaches [3,13,18].

Scientific evidence highlights exercise as an accessible, cost-effective, and viable therapeutic modality for managing most chronic pain conditions. Physical activity not only enhances cardiorespiratory function but also improves mental health and reduces pain [19,20]. The physiological mechanisms through which exercise exerts its analgesic effects are multifaceted.

One of the most well-supported mechanisms in the literature is exercise-induced hypoalgesia (EIH), which describes the capacity of physical exercise to activate descending inhibitory pain pathways at the central nervous system level [20,21], characterized by increased pain thresholds and tolerance, as well as reduced pain intensity ratings during and after exercise [20,22,23]. During and following exercise, there is an increased release of β-endorphins, serotonin, and endocannabinoids, which play a role in reducing pain perception both in peripheral tissues and in central structures such as the thalamus and insular cortex, contributing to pain modulation [24,25].

Additionally, muscle contractions can activate A-delta and C fibers in skeletal muscle, whose stimulation may lead to the activation of the endogenous opioid system [20,22,23]. This activates the descending modulatory pain systems (DMPs) that originate in the brainstem (e.g., the periaqueductal gray (PAG) and the rostral ventromedial medulla (RVM)), which exert both inhibitory and facilitatory effects on nociception. In states of chronic pain, these systems often present an imbalance, leading to persistent pain. Therefore, exercise represents a therapeutic strategy that may help restore this balance [20,22,23].

Moreover, it has been shown that exercise can reduce central sensitization, a common clinical finding in patients with chronic headaches, characterized by increased excitability of the central nociceptive system and a decreased pain threshold [26,27]. By increasing the pain threshold and improving cortical inhibitory function, exercise contributes to a reduction in the frequency and intensity of headache episodes, as it regulates brain-derived neurotrophic factor (BDNF). Although BDNF increases temporarily after an acute session of exercise, regular exercise can stabilize and often reduce BDNF levels in patients with chronic pain, thereby decreasing the hyperexcitability of nociceptors and central neurons [28,29].

The modulation of BDNF and glial cell activity by exercise provides a direct cellular mechanism to desensitize the central nervous system in patients with chronic headaches, suggesting that exercise functions not only as an analgesic but also as a disease-modifying intervention in chronic pain by directly addressing the underlying neuroplastic changes associated with central sensitization [30].

Another relevant mechanism is the modulation of the immune and neuroendocrine systems through a reduction in pro-inflammatory cytokines such as IL-1β, TNF-α, and IL-6 and an increase in anti-inflammatory cytokines such as IL-10, which play a key role in neurogenic inflammation associated with migraine [31]. This anti-inflammatory action, along with improved vagal tone and reduced sympathetic activity, allows for the regulation of autonomic dysfunction frequently observed in patients with chronic migraine [20,32].

The endocannabinoid system also plays an important role, as exercise increases circulating levels of endocannabinoids that interact with receptors involved in pain modulation [20]. Additionally, serotonergic pathways may interact with the opioid system to influence exercise-induced hypoalgesia [20]. The analgesic effects of exercise depend on a complex interaction among biological, psychological, and contextual factors [19,20].

Various exercise modalities, including running, walking, resistance training, aquatic exercise, and Tai Chi, have been studied for their effectiveness in alleviating chronic musculoskeletal pain, including chronic neck pain, osteoarthritis, fibromyalgia, and chronic lower back pain [12,19,33,34]. Given the increasing global prevalence of headache disorders, it is crucial to identify cost-effective strategies that improve both the functional and structural aspects of these pathologies.

While the benefits of exercise for chronic pain are recognized, there remains a gap in comprehensive, high-quality research specifically analyzing the direct effects of different exercise modalities on pain reduction in patients diagnosed with chronic tension-type headache or chronic migraine. There is a need for scientific evidence that explores tailored exercise prescriptions, their long-term efficacy, and their impact on central sensitization mechanisms in this particular patient population.

Hence, this study seeks to address the following research question: what is the effect of structured exercise interventions on pain intensity, headache frequency, and the modulation of central sensitization in individuals suffering from chronic tension-type headache or chronic migraine?

This systematic review aims to critically evaluate the efficacy of exercise interventions in reducing pain intensity and frequency in patients diagnosed with chronic tension-type headache or chronic migraine, with a particular focus on understanding the underlying physiological mechanisms of exercise’s impact.

## 2. Materials and Methods

This systematic review followed the criteria established by the PRISMA statement (Preferred Reporting Items for Systematic reviews and Meta-Analyses). The PICOS strategy (Patient, Intervention, Comparison, Outcome, Study design) (Table 1) was used, where P = patients with chronic tension-type headache or chronic migraine; I = exercise; C = conventional treatment; O = pain reduction; and S = RCTs and quasi-experimental trials [35,36]. The protocol of this study was published in the PROSPERO database, available at https://www.crd.york.ac.uk/PROSPERO/view/CRD420251027431, accessed on 16 April 2025.

### 2.1. Eligibility Criteria

#### 2.1.1. Inclusion Criteria

Randomized controlled trials (RCTs) and quasi-experimental trials were included, involving a sample of patients with tension-type headache or chronic migraine who received some type of exercise as an intervention, regardless of the country of origin or the language of the study. Additionally, other relevant studies identified through manual searching were also included.

#### 2.1.2. Exclusion Criteria

Systematic reviews, meta-analyses, observational studies such as case–control and cohort studies, case studies, and studies involving patients with pathologies of autoimmune origin, cancer, and uncontrolled respiratory and cardiovascular diseases were excluded. Furthermore, articles classified as having a high risk of bias according to the RoB 2 tool or with a score of <6 on the PEDro scale were excluded.

### 2.2. Information Sources

For the selection of articles meeting the inclusion criteria of this review, the following databases were chosen: PubMed, Web of Science, and Scopus. These were searched up to November 2024. 

### 2.3. Search Strategy

MeSH terms, Boolean operators (“AND”, “OR”), and filters for randomized controlled trials were used, when applicable, employing the following search strategies (Table 2).

### 2.4. Selection Process

All search results were organized, and duplicate records were removed using Mendeley. The selection process consisted of two phases. First, titles and abstracts were screened to identify relevant studies according to the eligibility criteria. Second, a comprehensive evaluation of the full texts of the selected articles was performed.

### 2.5. Data Extraction

All identified references were imported into the bibliographic software Mendeley Desktop to identify duplicates. Additionally, studies not retrieved in the initial search but meeting the inclusion and exclusion criteria of this systematic review—and extracted from the reference lists of eligible studies—were manually included. Subsequently, the articles were organized in a table according to their characteristics.

### 2.6. Data Items

1. For each included study, the following information was considered, which was distributed in two tables to facilitate the analysis and comprehension of the studies: general data of each study, including first author’s name, publication year, and country.
2. Study design and level of evidence: RCT or quasi-experimental. For the evaluation of the level of evidence, the CEBM scale (Centre for Evidence-Based Medicine) was considered.
3. Sample characteristics: Number of participants and diagnosis (chronic migraine, chronic tension-type headache, or both).
4. Intervention: Type of exercise, whether it was combined, and its training volume (weekly frequency, duration in minutes).
5. Control group: Conventional treatment, placebo, physiotherapy without exercise.
6. Findings: Clinical outcomes, along with the measurement instruments used.

Missing data not provided in the study were reported as N/A.

### 2.7. Study Risk of Bias Assessment

The PEDro scale (Physiotherapy Evidence Database scale), commonly used in randomized clinical trials (RCTs) and quasi-experimental studies, was used to assess the methodological quality of the studies [37] (Table A1). Additionally, the RoB 2 (Risk of Bias 2) assessment tool was applied to assess the risk of bias, specifically focusing on the effect of the intervention and categorizing the risk as low, slightly concerning, or high [38,39] (Table A2).

### 2.8. Synthesis Methods and Reporting Bias Assessment

The synthesis of the results obtained in this review, which were mainly qualitative, was carried out through the tabulation, summarization, and classification of information obtained from the studies meeting the inclusion criteria. This approach is necessary due to several factors.

There was significant heterogeneity in the characteristics of the interventions, including exercise types, frequencies, durations, and measurement instruments. Furthermore, variability exists in the patient populations and diagnoses, as the review encompassed studies focused solely on chronic tension-type headache (CTTH) and others that included mixed populations of CTTH and migraine (M). While these conditions share some mechanisms of central sensitization, they possess distinct pathophysiological profiles, such as the neurovascular component of migraine and the muscular component of CTTH. Although both are chronic headaches, this introduces significant clinical heterogeneity, implying that interventions effective for one might not be equally effective for the other, or their mechanisms of action could differ.

This means that even if an exercise appears beneficial for both conditions, the underlying reason for its efficacy might differ, or the magnitude of its effect could vary. Grouping them together would obscure these nuances and diminish the meaningfulness of a quantitative synthesis, as any measured “effect” could be an average across two distinct conditions. For these reasons, we decided to perform a narrative synthesis of the results, grouping studies based on the type of exercise applied and their main clinical outcomes.

The information was organized in tables to facilitate the interpretation of the results, the level of evidence, and the risk of bias for each study. To assess publication bias, the proportion of studies with positive results, the absence of negative findings, and the limited number of investigations in relation to the expected outcome for each were considered.

### 2.9. Evaluation of the Certainty of the Evidence

The certainty of the evidence was evaluated using the GRADE tool (Table 3), considering it moderate for the outcomes of pain intensity and frequency and low for quality of life due to the heterogeneity and small sample size.

## 3. Results

### 3.1. Study Selection Process

**Figure 1:**
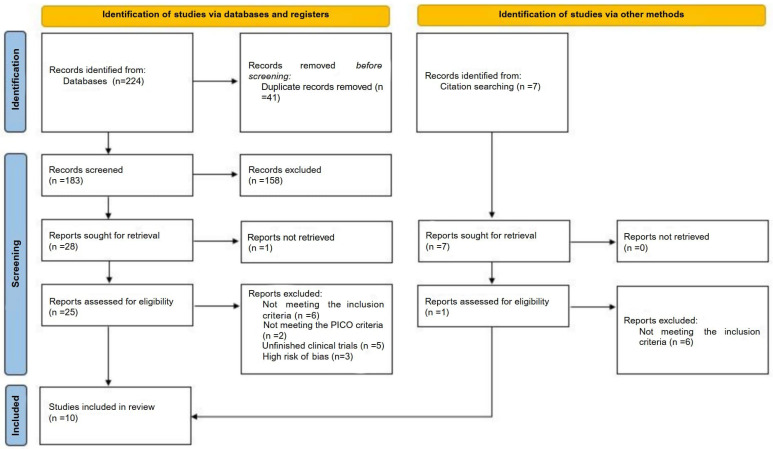
Flow diagram of included studies (PRISMA 2020 flow diagram for new systematic reviews, which included searches of databases, registers, and other sources).

In the initial search, 224 studies were identified: 35 in PubMed, 46 in Web of Science, and 143 in Scopus. After excluding studies that did not meet the eligibility criteria, a total of 10 studies were included in the review. The study selection process is detailed in Figure 1, which presents the flow diagram of the systematic review. The characteristics of the selected studies are presented in Table 4, Table 5, Table 6 and Table 7.

### 3.2. Characteristics of the Studies Included in the Systematic Review

The characteristics of the selected studies, which will be analyzed in the discussion, are presented in Table 4, Table 5, Table 6 and Table 7.

Ten studies were included in the review, all of which were RCTs except for one non-controlled trial, including eight studies with a type 1B level of evidence and two studies with a type 2B level of evidence according to the CEBM [40]. Six of them had two groups (one experimental and one control), and four were divided into multiple groups—one study had four subgroups (three experimental and one control), while the other three studies had two experimental groups and one control group each.

Of the ten clinical trials reviewed, the intervention groups included treatments with different types of exercise (strength, cardiovascular, resistance, relaxation, specific, or general), and five of them combined this with other types of therapeutic strategies. In contrast, the control groups in six studies received conventional care (medical treatment with ergonomic recommendations and medication), one group had a placebo treatment, and three were treated with a therapeutic technique, unlike the experimental group where the same technique was used as in the control group plus therapeutic exercise was performed.

The strategies used in the studies to address tension-type headache or migraine included medical care, relaxation exercises, soft tissue mobilization, acupuncture, ergonomic recommendations or pain management strategies, exercise, suboccipital muscle pressure inhibition, myofascial release, body awareness therapy, manual therapy, or strength training exercises.

According to the results found in the present review, the intervention period ranged from a minimum of 4 weeks to a maximum of 48 weeks. Regarding the exercise modality, the majority of interventions consisted of a strength and resistance training session, which included a warm-up with joint mobility or aerobic exercises, core training (elastic bands, isometrics, isotonics, etc.), and a cool-down with stretching exercises. Regarding the frequency, most exercises were performed three times per week, unlike four articles, where one was performed daily or two times a week, and the others were modified from one to two times a week or from two to three times a week. Session duration varied across studies, ranging from 15 to 60 min.

Regarding the findings and results, all studies reported a reduction in pain. Nine studies reported a significant decrease in pain intensity in the intervention groups that included exercise compared to controls. The remaining study showed improvements, although differences between groups were not statistically significant. Seven of the ten trials also reported a reduction in headache frequency in the intervention group compared to controls.

Furthermore, according to Table A1 (PEDro scale to evaluate the quality of the studies), four studies had a score of 8/10 and six scored 7/10, indicating good methodological quality. Table A2 (RoB 2 assessment tool to evaluate the risk of bias) indicated that all studies had some concerns regarding bias, primarily due to a lack of blinding of participants, therapists, and, in some cases, outcome assessors.

In this review, it was observed that most of the included studies reported favorable effects of physical exercise on pain intensity and frequency in patients with chronic headache. No studies with clearly negative or null results were identified, which may reflect a tendency to publish positive findings. Likewise, the scarcity of studies evaluating outcomes such as medication consumption or quality of life suggests a possible underrepresentation of less conclusive or unfavorable results.

Therefore, although the existence of publication bias cannot be confirmed with certainty, its presence cannot be ruled out, especially in relation to secondary outcomes. This limitation should be considered when interpreting the overall strength of the available evidence.

In the GRADE evaluation, it was identified that the studies related to pain intensity and frequency showed moderate certainty, due to their good methodological quality; however, they presented some heterogeneity and imprecision. In contrast, the results on quality of life and medication consumption showed low or very low certainty, due to the small number of studies, reduced sample size, and possible publication bias. These findings highlight the need for more homogeneous and methodologically rigorous research.

## 4. Discussion

This study aimed to answer the following research question: “What is the effect of structured exercise interventions on pain intensity, headache frequency, and the modulation of central sensitization in individuals suffering from chronic tension-type headache or chronic migraine?”

The findings from the reviewed literature consistently support that structured physical exercise can have positive effects on pain intensity and frequency in patients with CTTH and CM. These benefits were more pronounced when exercise was applied in a structured manner, with a minimum duration of four weeks and regular frequency.

However, it is crucial to prescribe the appropriate exercise intensity and frequency to achieve the desired hypoalgesic effects [51]. To do this, factors such as the type of exercise (aerobic exercise and resistance training) must be considered.

For aerobic exercise, scientific evidence recommends engaging large muscle groups to increase resting heart rate to the target heart rate for at least 20 min through repetitive muscle contractions [52]. Studies like Hoffman et al. [53] found that 30 min of treadmill exercise at 75% of VO_2_ max significantly decreased pain levels. Nevertheless, no significant changes were observed with 10 min of treadmill exercise at 75% of VO_2_ max or with 30 min at 50% of VO_2_ max [52]. This explains why the studies by Schiller et al. (2011) [41], Söderberg et al. (2011) [43], and Söderberg et al. (2006) [44] showed pain reduction effects with aerobic exercise exceeding 20 min.

Regarding resistance training, Koltyn [54] stated that resistance exercise can lead to a hypoalgesic response and that further studies were necessary to determine if a specific intensity was required to achieve hypoalgesia. Koltyn and Arbogast [55] discovered that a single 45 min session of resistance exercise produced an exercise-induced hypoalgesic response to resistance training [55].

In reference to specific versus general muscle work for exercise-induced hypoalgesia (EIH), it has been observed that the response is greater in the body part directly performing the exercise [13]. This finding supports why cervical stabilization exercises, as included in studies by Sung Hak Cho (2021) [42], Javdaneh et al. (2021) [50], Söderberg et al. (2011) [43], Söderberg et al. (2006) [44], Ylinen et al. (2005) [49], Martín-Vera et al. (2023) [46], and Park et al. (2024) [48], align with the results of the study by Vaegter et al. [56]. In the latter, participants performing aerobic exercise (cycling) at two intensities (low and high) and isometric exercises (arm and leg) at 30% and 60% of their maximal voluntary contraction showed greater EIH responses in the exercised body parts than in non-exercised parts across all exercise modalities. Pressure pain thresholds (PPTs) increased at both local and remote sites during both cold pressor tests under all exercise conditions. Furthermore, high-intensity exercise produced greater EIH responses than low-intensity exercise [56].

The studies in this review highlight the effectiveness of various exercise modalities. For instance, research by Martín-Vera et al. (2023) [46] and Ylinen et al. (2005) [49] underscore the benefits of resistance training [46,49].

Strength training, particularly focused on the stabilizing musculature of the cervical and scapular regions, has been shown to increase the pressure pain threshold in the neck musculature after several weeks of training. This leads to a reduction in cervical pain and associated disability [7,49]. Patients with chronic headaches often exhibit a high number of active myofascial trigger points, which arise from abnormal muscle activity and contribute to constant pain. Researchers like Sung Hak Cho (2021) [42] emphasize the importance of treating this affected musculature, complementing it with exercise and correcting postural habits. This comprehensive approach aims to increase the pain threshold, sustain treatment results, and prevent the recurrence of painful episodes [42]. Strength training focused on the neck and upper back, along with flexibility exercises, can reduce muscle tension, improve posture, and decrease the formation of myofascial trigger points that contribute to headache pain [46,57]. By alleviating biomechanical tension, the overload on pericranial and cervical muscles can be reduced, which is a key factor in the chronification of some headache types.

Beyond isolated exercise, many studies suggest that combining exercise with other interventions yields superior effects for decreasing symptoms in the short term and maintaining benefits over time. For example, Schiller et al. (2021) [41], Sung Hak Cho et al. (2021) [42], and Javdaneh et al. (2021) [50] observed better outcomes when exercise was integrated with other therapeutic approaches [41,42,50]. A notable example is Joerg Schiller’s study (2021) [41], which evaluated the effectiveness of acupuncture combined with exercise versus usual treatment in patients with tension-type headache. The intervention group receiving both strategies showed significant results [41]. Meanwhile, Soderberg et al. (2006) [44], who included acupuncture as an alternative to physical training, also found within-group improvements for both interventions, but the exercise group reported better outcomes in pain intensity and wellbeing [41,44]. The positive effects of exercise on chronic headache are consistent with the understanding that exercise improves central pain modulation, a crucial factor in the pathophysiology of chronic headache [58,59]. The efficacy of exercise in managing chronic headaches is intricately linked to its influence on central pain mechanisms, central sensitivity syndromes (CSSs), and central nervous system (CNS) sensitization. Regular exercise can modulate these neural circuits, improving the brain’s ability to inhibit pain through descending pain modulation systems [57,60]. These pathways release neurotransmitters such as serotonin and norepinephrine, which can suppress the transmission of pain signals [19,20]. This is fundamental to breaking the cycle of chronic pain.

Moreover, regular exercise, especially aerobic types, similar to those investigated in the training program by Ylinen et al. (2005) [49], which demonstrated positive effects on pain reduction, stimulates the release of endorphins, the body’s natural painkillers. These interact with opioid receptors in the brain, decreasing pain perception and promoting a sense of wellbeing. Furthermore, regular exercise, especially aerobic types, influences the release of other neurotransmitters like serotonin and dopamine, which play a crucial role in pain and mood regulation and are often dysregulated in patients with chronic headache [5,6]. Physical activity also stimulates the release endocannabinoids, which are natural pain-relieving compounds. These substances act on pain receptors in the brain and spinal cord, contributing to exercise-induced analgesia [19]. Moreover, chronic pain states often involve low-grade neuroinflammation in the CNS. In particular, aerobic exercise has anti-inflammatory effects, both peripherally and centrally. It can reduce the release of pro-inflammatory cytokines and increase anti-inflammatory mediators, thereby dampening the inflammatory processes that contribute to central sensitization [20].

Furthermore, studies by Soderberg et al. (2006) [44], Gopichadran et al. (2024) [45], and Loew et al. (2000) [47] have reported positive results from relaxation exercises combined with breathing techniques, good posture, or body awareness [44,45,47]. These approaches are likely effective due to their combined impact on the psycho-physical alterations involved in the etiology of migraine and TTH. They reduce peripheral muscle tension in the head and neck regions, and simultaneously, relaxation techniques alleviate general muscle tension and excessive activation, thereby improving central pain control mechanisms. Chronic headache is often linked to the dysregulation of the autonomic nervous system, with an imbalance favoring sympathetic over parasympathetic activity. Exercise, especially modalities incorporating relaxation and breathwork, can help restore autonomic balance, reducing overall physiological arousal and muscle tension, which can exacerbate headaches [1].

The potential of body-oriented strategies like postural integration and therapeutic yoga, as proposed by Sertel et al. (2017) [61] and Loew et al. (2000) [47], further reinforces this integrated approach [47,61]. Chronic pain is associated with maladaptive neuroplastic changes in the brain, including alterations in cortical representation. This type of regular exercise can induce beneficial neuroplasticity, potentially normalizing brain activity patterns related to pain processing [20,22,23,62,63,64].

Beyond direct physiological effects, exercise profoundly impacts psychological factors often co-occurring with chronic headaches, such as stress, anxiety, and depression. By improving mood, self-efficacy, and coping strategies, exercise indirectly reduces the impact of these factors on pain perception and central sensitization [19,20,22,23].

### 4.1. Strengths and Limitations

The review followed the PRISMA protocol, applying tools for assessing the risk of bias and a structured narrative synthesis. However, the main limitations were the heterogeneity of the interventions, the variability of the instruments used, and the lack of blinding in several studies.

Regarding RCT-type studies that relate the hypoalgesic effect of exercise and chronic tension-type headache and chronic migraine, despite having carried out a broad search in the databases most used by physiotherapists, it was only possible to identify 10 articles. This is an area open to future research, as most of the scientific evidence is focused on lower back pain and fibromyalgia.

Another of the main limitations found in the search was related to the scarce evidence that addresses treatment in chronic patients with tension-type headache and migraine, since most of the studies are related to episodic primary headaches. Furthermore, there is little quality scientific evidence that considers aerobic exercise in tension-type headache, unlike migraine, and the studies that do consider it have a moderate or high risk of bias due to the difficulty of blinding patients, therapists, and in some cases, evaluators as well.

Although more scientific evidence of exercise’s effect on migraine has been found in the last two years, it is almost impossible to find completed studies or studies with a low risk of bias that relate exercise to chronic migraine, which made it difficult to collect information in the review, and therefore only two studies with mixed patients as a sample were found.

The variability of the interventions makes it impossible to determine the best exercise modality or the specific protocol for each subgroup of patients.

### 4.2. Clinical Implications and Future Lines of Research

The evidence suggests that physical exercise does not merely act as a symptomatic reliever but engages complex physiological mechanisms that directly address the core pathology of central sensitization in chronic headache conditions. This deeper understanding underscores the importance of integrating structured exercise into comprehensive management plans for individuals suffering from CTTH and CM.

Physical exercise should be considered a complementary therapeutic strategy for the treatment of chronic headache and migraine. It is recommended that future research compare different types of exercise, evaluate the long-term effect, and include standardized measures of quality of life and adherence.

## 5. Conclusions

Exercise can significantly decrease the intensity and frequency of headache episodes in patients with chronic migraine and chronic tension-type headache, considering that most of the included studies of good quality according to the PEDro scale (with a level of evidence of 1B–2B) in this review reported positive effects in at least one of the mentioned variables.

However, the heterogeneity of the studies and the moderate risk of bias due to the difficulty or impossibility of blinding the sample and the therapists limit the strength of this statement, for which we can say that the evidence is reasonably reliable and that it requires confirmation by studies with greater methodological rigor.

To obtain the hypoalgesic effect, it is recommended to combine aerobic exercise that is best suited to the individual’s needs with postural re-education activities that allow the individual to maintain correct posture and a therapeutic strategy that decreases the tension of adjacent soft tissues such as relaxation exercises, massage, stretching, or dry needling, since tension-type headache and migraine, as evidenced in this review, are of multifactorial origin.

## Figures and Tables

**Table 1:** The PICOS strategy.

PICO Question: Is Exercise Effective in Reducing Pain in Patients with Chronic Tension-Type Headache and Chronic Migraine?				
Patient or Population	Intervention	Comparison	Outcome	Study type
Patients with Chronic Tension-Type Headache	Aerobic exercise Anaerobic exercise Exercise	Conventional treatment. Physiotherapy treatment without exercise Placebo	Pain reduction Hypoalgesia	RCT studies Quasi-experimental clinical trials
Patients with Chronic Migraine				

**Table 2:** Search strategies.

Search Strategies	
Database	Search Criteria or Terms
PubMed	Search: chronic tension headache AND exercise OR chronic migraine AND exercise Filters: Clinical Trial, Randomized Controlled Trial Sort by: Computed Author ((((“chronic”[All Fields] OR “chronical”[All Fields] OR “chronically”[All Fields] OR “chronicities”[All Fields] OR “chronicity”[All Fields] OR “chronicization”[All Fields] OR “chronics”[All Fields]) AND (“tension type headache”[MeSH Terms] OR (“tension type”[All Fields] AND “headache”[All Fields]) OR “tension type headache”[All Fields] OR (“tension”[All Fields] AND “headache”[All Fields]) OR “tension headache”[All Fields]) AND (“exercise”[MeSH Terms] OR “exercise”[All Fiel ds] OR “exercises”[All Fields] OR “exercise therapy”[MeSH Terms] OR (“exercise”[All Fields] AND “therapy”[All Fields]) OR “exercise therapy”[All Fields] OR “exercising”[All Fields] OR “exercise s”[A ll Fields] OR “exercised”[All Fields] OR “exerciser”[All Fields] OR “exercisers”[All Fields])) OR ((“chronic”[All Fields] OR “chronical”[All Fields] OR “chronically”[All Fields] OR “chronicities”[All Fields] OR “chronicity”[All Fields] OR “chronicization”[All Fields] OR “chronics”[All Fields]) AND (“migrain”[All Fields] OR “migraine disorders”[MeSH Terms] OR (“migraine”[All Fields] AND “disorders”[All Fields]) OR “migraine disorders”[All Fields] OR “migraine”[All Fields] OR “migraines”[All Fields] OR “migraine s”[All Fields] OR “migraineous”[All Fields] OR “migrainers”[All Fields] OR “migrainous”[All Fields]))) AND (“exercise”[MeSH Terms] OR “exercise”[All Fields] OR “exercises”[All Fields] OR “exercise therapy”[MeSH Terms] OR (“exercise”[All Fields] AND “therapy”[All Fields]) OR “exercise therapy”[All Fields] OR “exercising”[All Fields] OR “exercise s”[All Fields] OR “exercised”[All Fields] OR “exerciser”[All Fields] OR “exercisers”[All Fields])) AND (clinicaltrial[Filter] OR randomizedcontrolledtrial[Filter])
Web of Science	Search chronic tension headache AND exercise OR chronic migraine AND exercise (Topic) and Preprint Citation Index (Exclude—Database) and Clinical Trial (Document Types) (TS = (chronic tension headache AND exercise OR chronic migraine AND exercise)) AND ((DT==(“CLINICAL TRIAL”)) NOT (SILOID==(“PPRN”)))
Scopus	(TITLE-ABS-KEY(“chronic tension headache” AND exercise) OR TITLE-ABS-KEY(“chronic migraine” AND exercise)) AND (LIMIT-TO (EXACTKEYWORD,”Tension Headache”) OR LIMIT-TO (EXACTKEYWORD,”Migraine”) OR LIMIT-TO (EXACTKEYWORD,”Exercise”)) AND (LIMIT-TO (DOCTYPE,”ar”) OR LIMIT-TO (DOCTYPE,”re”))

**Table 3:** Certainty assessment GRADE tool.

GRADE Evidence Profile—Summary of Findings						
Outcome	Risk of Bias	Inconsistency	Imprecision	Indirect Evidence	Publication Bias	GRADE Certainty
Pain intensity	Moderate	Moderate	Low	No	Possible	Moderate
Headache frequency	Moderate	Moderate	Moderate	No	Possible	Moderate
Quality of life	High	High	High	Moderate	Probable	Low
Medication use	High	High	High	High	Probable	Very low

**Table 4:** Characteristics of the studies included in this systematic review of studies with patients with chronic tension-type headache.

Characteristics of the Studies Included in the Systematic Review (Chronic Tension-Type)								
Author	Year	Country	Study Type	Level of Evidence (CEBM) [40]	Sample	Intervention	Control	Most Relevant Findings
Schiller et al. [41]	2021	Germany	RCT	1B	96 (CTTH)	Group 2 (*n =* 24): acupuncture (AP), Group 3 (*n =* 24): Medical Training Therapy (MTT), Group 4 (*n =* 24): acupuncture + exercise (AP + MTT).	Group 1 (*n =* 24): Usual care (UC).	The combination of acupuncture and exercise was significantly superior in reducing pain intensity compared to usual care (*p* = 0.012), from baseline to 3-month follow-up (T0–T3) and to 6-month follow-up (T0–T4).
Sung Hak Cho [42]	2021	Korea	RCT	2B	45 (CTTH)	Group A or SMI (*n =* 15): Inhibition of suboccipital muscles and myofascial release technique (MFR). Group B or SMIEx (*n =* 15): Myofascial release technique (MFR) and postural correction exercises (FHP).	Group C (*n =* 15): Control (no intervention).	Comparison with the control group at the end of the intervention: HIT-6 decreased significantly only in the SMIEx group (*p <* 0.05), while headache PPT increased significantly only in the SMIEx group (*p <* 0.05). aTrP also showed a significant reduction in both the SMI and SMIEx groups (*p <* 0.05). However, lTrP showed no significant difference.
Söderberg et al. [43]	2011	Sweden	RCT	1B	90 (CTTH)	Relaxation training group (*n* = 30) involves focusing on the sensation of relaxation of the body, stress coping, and how to relax in daily life. Physical training group (*n* = 30): The treatment lasted 3 months with 25 training sessions of 45 min, 3 times per week.	Acupuncture group (*n* = 30): The treatment consisted of 10 to 12 sessions over a period of 10 to 12 weeks.	All treatments proportionally improved subjective symptoms related to the central nervous system in patients with chronic CT compared to baseline. At the 3-month follow-up, the total score of the minor symptom evaluation profile improved significantly in the physical training group compared to the acupuncture group (*p <* 0.01). Vitality and sleep improved significantly at the 6-month follow-up in the relaxation training group compared to the acupuncture group (*p <* 0.005).
E. Söderberg et al. [44]	2006	Sweden	RCT	1B	90 (CTTH)	Relaxation training group (*n* = 30). Physical training group (*n* = 30).	Acupuncture group (*n* = 30).	The relaxation group reported a significantly greater number of headache-free periods (*p* < 0.05) and a significantly greater number of headache-free days (*p* < 0.01) compared to the acupuncture group immediately after the last treatment. There were no other significant differences between the study groups at any time.
Gopichandran et al. [45]	2024	India	RCT	1B	169 (CTTH)	Experimental group (*n =* 84): PMR (Progressive Muscle Relaxation) and deep breathing exercises (+4 physiotherapy sessions of massage and mobilization)	Control group (*n =* 85): Standard routine care (without specific interventions but with 4 physiotherapy sessions of massage and mobilization).	PMR therapy and deep breathing exercises effectively reduced pain intensity and frequency according to the Wong–Baker scale (*p =* 0.001), along with headache-related disability according to HIT-6 (*p <* 0.001), and improved sleep quality (*p <* 0.001) at 4, 8, and 12 weeks compared to the control group.
Martín-Vera et al. [46]	2023	Spain	RCT	1B	40 (CTTH)	Intervention group (*n =* 20): Strength exercises.	Control group (*n =* 20): Conventional treatment.	The intervention group showed statistically significant differences related to the decrease in the duration (*p =* 0.007) and intensity of headaches (*p =* 0.001), increased thickness of the deep cervical muscles (*p <* 0.001), reduced peripheral sensitization (*p <* 0.05), and improved strength of the deep cervical flexors (*p <* 0.001) compared to the control group at the end of the study. There were no significant differences in cervical ROM except for inclination.
Loew et al. [47]	2000	Germany	RCT	1B	54 initially and 36 at the end (CTTH)	eFR group (elements of FR or functional relaxation) (*n =* 27). This is a relaxation technique that combines gentle movements, breathing, and concentration on body perception to reduce muscle tension and promote relaxation. At the end of the study, *n =* 24.	Placebo group (*n =* 27) with UIR (Non-specific Intervention Technique) based on isotonic exercises of one hand (without involving the neck muscles). At the end of the study *n =* 12.	In the functional relaxation group (eFR), a significant reduction was found in the sum of total hours of pain and in the intensity (high and medium) with respect to the UIR (Non-specific Intervention Technique) at the end of the study. There was a reduction in intense pain (level 3; *p* = 0.003) in the eFR group, as well as a significantly greater reduction in moderate pain (level 2; *p* = 0.03). In addition, the eFR group presented significantly fewer total days of pain (*p* = 0.04). All these findings were compared with the control group post-treatment.
Park et al. [48]	2024	South Korea	RCT	2B	40 (CTTH)	Experimental group (*n =* 20): Manual therapy, stabilization exercises, and eye exercises.	Control group (*n =* 20): Manual therapy and stabilization exercises.	Both groups showed significant improvements in NRS (Numerical Rating Scale) scores, NDI (Neck Disability Index), SF-12 (Short Form-12 health survey questionnaire) and HIT-6 (Headache Impact Test-6), CVA (craniovertebral angle), CRA (cranial rotation angle), and muscle tone (*p <* 0.05). The experimental group presented significant differences in the scores of NDI, SF-12, and HIT-6 and in suboccipital muscle tone compared to the control group (*p <* 0.05).

HIT-6: Headache Impact Test; NPAD: Northwick Park Neck Pain Questionnaire; PPT: pain pressure threshold; SF-12: 12-item Short Form Health Survey; CTTH: chronic tension-type headache; CM: chronic migraine; TTH: tension-type headache; PNE: pain neuroscience education; GI: intervention group; GC: control group; T0: baseline; T3: follow-up No. 3 (3 months); T4: follow-up No. 4 at 6 months; AP: acupuncture; MTT: Medical Training Therapy; SMI: myofascial release technique; MFR: myofascial release; SMIEx: myofascial release technique and postural correction exercises; FHP: postural correction exercises; PMR: progressive muscle relaxation; FR: functional relaxation; eFR: elements of functional relaxation; UIR: Non-Specific Intervention Technique; TrP: myofascial trigger point; aTrP: active myofascial trigger point; lTrP: latent myofascial trigger point; PCS: Pain Catastrophizing Scale; FABQ: Fear-Avoidance Beliefs Questionnaire; PSEQ: Pain Self-Efficacy Questionnaire; VAS: Visual Analog Scale; ROM: range of motion; NRS: Numerical Pain Rating Scale; NDI: Neck Disability Index; CVA: craniovertebral angle; CRA: cranial rotation angle.

**Table 5:** Characteristics of the studies included in the systematic review (of studies with patients with chronic tension-type headache and chronic migraine).

Characteristics of the Studies Included in the Systematic Review (Chronic Tension-Type Headache and Chronic Migraine)								
Author	Year	Country	Study Type	Level of Evidence (CEBM) [40]	Sample	Intervention	Control	Most Relevant Findings
Ylinen et al. [49]	2005	Finland	RCT	1B	180 (CTTH and CM)	Training groups: Resistance (*n =* 59) and strength (*n =* 60).	Control group (*n =* 60): 4 physiotherapy sessions that included massage and mobilization.	Both resistance and strength training of the neck and shoulder muscles produced higher pressure pain threshold values in both training groups compared to the control group during the 12-month follow-up (*p <* 0.001).
Javdaneh et al. [50]	2021	Switzerland	RCT	1B	62 (CTTH and CM)	Therapeutic exercise-only group (*n* = 24) and combined group (therapeutic exercises + Pain Neuroscience Education Program: PNE) (*n* = 24).	Control group (*n =* 24), ergonomic recommendations at work and home.	For all measured variables, the effects of both exercise alone and the combined group (exercise + pain neuroscience education) were significantly superior compared to the control group (*p* < 0.05) at the end of the study.

CTTH: chronic tension-type headache; CM: chronic migraine; TTH: tension-type headache; PNE: pain neuroscience education.

**Table 6:** Intervention and outcomes in chronic tension headache.

Intervention and Outcomes in Chronic Tension Headache							
Study	Type of Exercise	Combined with Another Intervention	Intervention Duration	Frequency per Week	Duration in Minutes	Assessment Instruments	Results
Schiller et al., 2021 [41]	- Cardiovascular - Strength resistance - Coordination - Proprioception - Mobility and flexibility	Yes	6 weeks	1 to 2	35 min.	- Pain intensity - Frequency of headache days - Duration of pain episodes - Use of headache medication	In all groups, the frequency of headache days per month was significantly reduced from baseline (T0) to T3 (*p* = 0.76) and T4 (*p* = 0.62). - In all groups, the duration of pain episodes and the use of headache medication were substantially reduced between T0 and T3 (*p* duration = 0.59; *p* medication = 0.18).
Sung Hak Cho, 2021 [42]	- Postural correction exercises with light resistance for head flexors. - Pull-ups - Stretches	Yes	4 weeks	2	15 min	- HIT-6 - Pain pressure threshold (PPT) - Measurement of myofascial trigger point (active/latent TrP)	In the intragroup analysis, HIT-6 and headache PPT had significant changes on the left side in the SMI group (*p <* 0.05), while in the SMIEx group, the active myofascial trigger point (aTrP) showed a significant reduction in the HIT-6 group and in the SMIEx group with left and right headache PPT (*p <* 0.05).
E. I. Söderberg et al., 2011 [43]	Physical training: - Warm-up - Strengthening of cervical muscles and upper limbs - Cycling - Stretching	No	12 weeks (3 months)	3	45 min	- Minor Symptom Evaluation Profile (MSEP) questionnaire.	No differences were observed in the MSEP at the end of treatment between the three groups, nor at the 6-month follow-up; however, the 3-month follow-up was significantly higher in the physical training group (*p* = 0.036).
E. Söderberg et al., 2006 [44]	Physical training: - Warm-up - Strengthening of cervical muscles and upper limbs - Cycling - Stretching	No	12 weeks (3 months)	3	45 min	- Pain intensity - Frequency of pain - Free days	In the three groups (acupuncture, physical training, and relaxation), headache intensity decreased significantly both 3 and 6 months after the last treatment compared to the baseline value. However, headache-free days and pain-free periods increased immediately after, and at 3 and 6 months after the last treatment in the training and relaxation groups, the acupuncture group had no significant differences.
Gopichandran et al., 2024 [45]	Relajación muscular progresiva (PMR) y ejercicios de respiración profunda; La PMR se define como estiramiento y relajación voluntaria de un grupo muscular	Yes	12 weeks (3 months)	N/A	20 min	- Pain (Wong–Baker scale) - Disability (HIT-6) - Sleep quality (Pittsburgh Sleep Quality Index)	There was no significant difference in headache frequency between the groups (*p =* 0.109). - The intervention group (IG) significantly reduced the duration of headaches (*p =* 0.007) compared to the control group (CG) at the end of the study. - The IG significantly reduced intensity (*p =* 0.001) compared to the CG at the end of the study. - Increase in the thickness of the right multifidus muscle (contracted (*p <* 0.001), right deep flexors contracted (*p* < 0.001), and left deep flexors contracted *p* = 0.003)) of the IG compared to CG at the end of the study. - The IG had significantly increased strength (*p <* 0.001) compared to the CG. - There were no significant differences in cervical ROM except for inclination between the IG and CG. - The IG significantly improved PPT of the temporal (*p <* 0.001), right trapezius (*p =* 0.047), and left trapezius (*p =* 0.022) compared to the CG at the end of the intervention.
Martín-Vera et al., 2023 [46]	Therapeutic exercise: Strengthening exercises for cranio-cervical musculature, shoulder girdle, and shoulder (warm-up through joint mobility, shoulder exercises with TheraBand and isometrics for cervical musculature with manual resistance).	No	12 weeks (3 months)	- 2 (the first 6 weeks) - 3 (remaining 6 weeks)	N/A	- Headache duration (hours/day) - Pain intensity (VAS) - Frequency of episodes (days/month) - Muscle thickness (ultrasonography) - Cranio-cervical flexion test (CCFT) for functionality - Cervical stabilizer (20 to 30 mmHg) to assess strength. - ROM (CROM) - Algometer (PPT: pain pressure threshold) del dolor	Regarding the comparison of pre- and post-intragroup values, the control group did not present significant differences, the intervention group presented, in the primary results, a significant difference in the duration of headaches (*p =* 0.007) and intensity of headaches on the VAS (*p =* 0.001).
Loew et al., 2000 [47]	Functional relaxation (FR) exercises: This is a relaxation technique that combines gentle movements, breathing, and concentration on body perception to reduce muscle tension and promote relaxation.	No	8 weeks (approximately 60 days, 2 months)	Every day	45 min (1 or 2 times per hour)	German headache diary (followed the IHS criteria in relation to the ASTRA headache diary)	The intragroup analysis from the beginning to the end of the investigation did not show significant differences.
Park et al., 2024 [48]	- Cervical stabilization exercise with pressure biofeedback (method described by Abdel et al., modified). - Eye exercise program (method described by Sakshi and Kumar with modification). The method seeks to use eye movements to relax the muscles of the periocular region.	Yes	6 weeks (1.5 months)	3	- 30 min - 50 min	- NRS, NDI, SF-12, and HIT-6 assessments - Comparison of CRA and CVA - Muscle tone of the suboccipital and upper trapezius muscles	- Both groups showed significant changes (*p* < 0.05). Variations in CVA and CRA did not differ significantly between both groups (*p* > 0.05). - In addition, a significant difference was observed between both groups in the variation in muscle tone of the suboccipital muscle (*p* < 0.05)

Pre: pre-intervention: Post: post-intervention; HIT-6: Headache Impact Test; PPT: pain pressure threshold; TrP: myofascial trigger point; aTrP: active myofascial trigger point; lTrP: latent myofascial trigger point; NPAD: Northwick Park Neck Pain Questionnaire; PCS: Pain Catastrophizing Scale; FABQ: Fear-Avoidance Beliefs Questionnaire; PSEQ: Pain Self-Efficacy Questionnaire; VAS: visual analog scale; ROM: range of motion; NRS: Numerical Pain Rating Scale; NDI: Neck Disability Index; SF-12: Short Form Health Survey; CVA: craniovertebral angle; CRA: cranial rotation angle; SMI: myofascial release technique; MFR: myofascial release; SMIEx: myofascial release technique and postural correction exercises; FHP: postural correction exercises; PMR: progressive muscle relaxation; FR: functional relaxation; eFR: elements of functional relaxation; MSEP: Minor Symptom Evaluation Profile Questionnaire.

**Table 7:** Intervention and results of studies with patients with chronic tension-type headache and chronic migraine.

Intervention and Outcomes in Chronic Tension Headache and Chronic Migraine							
Study	Type of Exercise	Combined with Another Intervention	Intervention Duration	Frequency per Week	Duration in Minutes	Assessment Instruments	Results
Javdaneh et al., 2021 [50]	Therapeutic exercise (warm-up, strength exercises, resistance of neck, scapula and arm muscles, and cool-down exercises)	Yes	6 weeks	3	30–40 min (10 warm-up, 15–20 min exercise, 10 min cool-down)	- Neck Pain and Disability Scale (NPAD) - Pain Catastrophizing Scale (PCS) - Fear-Avoidance Beliefs Questionnaire (FABQ) - Pain Self-Efficacy Questionnaire (PSEQ)	PNE + therapeutic exercises led to a greater reduction in the pain disability index (*p <* 0.001), fear-avoidance beliefs (*p =* 0.041), and pain catastrophizing (*p =* 0.99) compared to therapeutic exercise alone at the end of the study. For pain self-efficacy, there were no statistically significant differences between the two intervention groups; however, the combined group had a more significant effect on increasing self-efficacy (*p* = 0.99)
Ylinen et al., 2005 [49]	- Resistance training - Strength training	Yes	48 weeks (12 months)	3	Between 45 and 60 min	- Algometry (PPT) - VAS (Visual Analog Scale)	There was no significant difference in PPT between the training groups. - Reduction (VAS) of 69% in the strength training group, 61% in the resistance training group, and 28% in the control group compared to baseline values.

PPT: pain pressure threshold; NPAD: Northwick Park Neck Pain Questionnaire; PCS: Pain Catastrophizing Scale; FABQ: Fear-Avoidance Beliefs Questionnaire; PSEQ: Pain Self-Efficacy Questionnaire; VAS: Visual Analog Scale.

## Data Availability

The original contributions presented in the study are included in the article, and further inquiries can be directed to the corresponding author.

## Abbreviations

The following abbreviations are used in this manuscript:

TTH	Tension-type headache
CSS	Central sensitivity syndrome
CNS	Central nervous system
CS	Central sensitization
CTTH	Chronic tension-type headache
CM	Chronic migraine

## Appendix A

**Table A1:** PEDRo scale.

PEDro Scale												
Study	Eligibility Criteria	Random Allocation	Allocation Concealment	Baseline Similarity	Blinding of Subjects	Blinding of Therapists	Blinding of Assessors	Adequate Follow-Up	Intention-to-Treat	Between-Group Comparisons	Measures of Variability	Total
Schiller et al., 2021 [41]	Yes	Yes	Yes	Yes	No	No	Yes	No	Yes	Yes	Yes	7/10
Sung hak cho, 2021 [42]	Yes	Yes	Yes	Yes	No	No	Yes	Yes	Yes	Yes	Yes	8/10
Javdaneh et al., 2021 [50]	Yes	Yes	Yes	Yes	No	No	Yes	Yes	Yes	Yes	Yes	8/10
E. I. Söderberg et al., 2011 [43]	Yes	Yes	Yes	Yes	No	No	No	Yes	Yes	Yes	Yes	7/10
E. Söderberg et al., 2006 [44]	Yes	Yes	Yes	Yes	No	No	No	Yes	Yes	Yes	Yes	7/10
Ylinen et al., 2005 [49]	Yes	Yes	Yes	Yes	No	No	Yes	Yes	Yes	Yes	Yes	8/10
Gopichandran et al., 2024 [45]	Yes	Yes	Yes	Yes	No	No	No	Yes	Yes	Yes	Yes	7/10
Martín-Vera et al., 2023 [46]	Yes	Yes	Yes	Yes	No	No	Yes	Yes	Yes	Yes	Yes	8/10
Loew et al., 2000 [47]	Yes	Yes	Yes	Yes	Yes	No	No	No	Yes	Yes	Yes	7/10
Park et al., 2024 [48]	Yes	Yes	Yes	Yes	No	No	No	Yes	Yes	Yes	Yes	7/10

**Table A2:** RoB 2 scale.

RoB 2 Scale						
Study	Randomization Process	Deviations from Intended Interventions	Missing Outcome Data	Measurement of the Outcome	Selection of the Reported Result	Overall Judgment
Schiller et al., 2021 [41]	Low	Some concerns	Low	Low	Low	Some concerns
Sung Hak Cho, 2021 [42]	Low	Some concerns	Low	Low	Low	Some concerns
Javdaneh et al., 2021 [50]	Low	Low	Low	Low	Low	Low
E. I. Söderberg et al., 2011 [43]	Low	Some concerns	Low	Some concerns	Low	Some concerns
E. Söderberg et al., 2006 [44]	Low	Some concerns	Low	Some concerns	Low	Some concerns
Ylinen et al., 2005 [49]	Low	Some concerns	Low	Some concerns	Low	Some concerns
Gopichandran et al., 2024 [45]	Low	Some concerns	Low	Some concerns	Low	Some concerns
Martín-Vera et al., 2023 [46]	Low	Some concerns	Low	Low	Low	Some concerns
Loew et al., 2000 [47]	Low	Some concerns	Some concerns	Low	Low	Some concerns
Park et al., 2024 [48]	Low	Some concerns	Low	Low	Low	Some concerns
